# Cellular Automaton Mimicking Colliding Bodies for Topology Optimization

**DOI:** 10.3390/ma15228057

**Published:** 2022-11-15

**Authors:** Bogdan Bochenek, Katarzyna Tajs-Zielińska

**Affiliations:** Faculty of Mechanical Engineering, Cracow University of Technology, 31-155 Krakow, Poland

**Keywords:** topology optimization, cellular automaton, colliding bodies, heuristic update rules

## Abstract

Needs and demands of contemporary engineering stimulate continuous and intensive development of design methods. Topology optimization is a modern approach which has been successfully implemented in a daily engineering design practice. Decades of progress resulted in numerous applications of topology optimization to many research and engineering fields. Since the design process starts already at the conceptual stage, innovative, efficient, and versatile topology algorithms play a crucial role. In the present study, the concept of the original heuristic topology generator is proposed. The main idea that stands behind this proposal is to take advantage of the colliding bodies phenomenon and to use the governing laws to derive original Cellular Automata rules which can efficiently perform the process of optimal topologies generation. The derived algorithm has been successfully combined with ANSYS, a commercial finite element software package, to illustrate its versatility and to make a step toward engineering applications. Based on the results of the tests performed, it can be concluded that the proposed concept of the automaton mimicking colliding bodies may be an alternative algorithm to other existing topology generators oriented toward engineering applications.

## 1. Introduction

As it has been observed over the years, topology optimization has been a dynamically developing research area with numerous applications to many research and engineering fields. The researchers community continuously works on innovative, efficient, and versatile topology optimization approaches, methods, and algorithms, whereas the spectrum of numerous solutions of topology optimization problems ranges from classic Michell structures to sophisticated contemporary engineering ones. The various approaches to the generation of optimal topologies have been presented along with emerging concepts which have been implemented in a broadly understood engineering area. The comprehensive discussion on various aspects of topology optimization has been provided by many survey papers: e.g., [1,2,3,4] recently complemented by Ribeiro et al. [5] and Logo and Ismail [6]. The long-lasting development of topology optimization confirms that it still remains one of the most important research fields within the area of structural and material design.

Along with the research issues of topology optimization, the practical aspects of engineering implementation of topology optimization techniques have become more and more important. As a result, the topology optimization tools are nowadays present in commercial engineering software. However, the black-box topology generators implemented into commercial software do not guarantee that the final results are the best available. Therefore, although remarkable achievements have been already made toward topology optimization application in engineering, there is still room for further investigations. Recently published papers [7,8,9,10,11] may serve here only as examples.

In the present study, the concept of the original heuristic topology generator is proposed. The main idea that stands behind this proposal is to take advantage of the colliding bodies phenomenon, and use the governing laws to derive original Cellular Automata rules which can efficiently perform optimal topologies generation process. The inspiration for this proposal was the series of papers by Kaveh and co-workers [12,13,14,15] in which the concept of Colliding Bodies Optimization for a function minimization has been proposed. This paper proposes an original technique which is also inspired by the collision of bodies phenomenon but this time it is oriented toward optimization of structure topology. It is worth underlining that the rules are built so as to cope also with irregular finite element meshes. The derived algorithm has been combined with ANSYS 14.0, a commercial finite element software package, to illustrate its versatility.

As mentioned above, the applied approach is based on the concept of Cellular Automata (CA). Cellular Automata are built so as to mimic the behavior of complicated systems in a relatively easy way. From a computational point of view, the special local rules are implemented with a view to control the performance of a system under consideration. Hence, the local physical quantities are respectively updated, which allows us to describe the global behavior of the system. The concept of Cellular Automata has been known since the late 1940s when von Neumann and Ulam proposed this idea. Henceforth, this approach has been found interesting by researchers representing various fields but probably for the first time topology optimization has been discussed within the CA approach only in the paper by Inou et al. [16]. Many papers have been hereafter published on that subject, and the majority of them have appeared during the last two decades, see e.g., [17,18,19,20] or [21]. The efficient CA algorithm has been also proposed and then developed by Bochenek and Tajs-Zielińska [22,23] and recently [24,25].

The outline of the paper is as follows. In Section 2, the topology optimization problem is formulated, then the concept of Cellular Automata mimicking colliding bodies is introduced, and finally the algorithm built based on this idea is described. Its implementation in the topology generation process is illustrated by an introductory example. Original examples of topology generation of selected 2D structures are discussed in the first part of Section 3 presenting performance of the topology generator. Next in this section, utilizing results of the preliminary computations, the Cellular Automaton is combined with ANSYS as the efficient structural analysis tool and its application to selected, both plane and spatial, engineering tasks is presented. With a view to cover a broad area of implementations, the discussed tasks include also irregular cell lattice. Based on the results of performed tests, the paper ends with concluding remarks in Section 4.

## 2. Methods and Concepts

In this section, the topology optimization problem is formulated, the concept of Cellular Automata mimicking colliding bodies is introduced, and the algorithm built based on this idea is described. The introductory example illustrates the implementation of the proposed concept into the topology generation process.

### 2.1. Structural Topology Optimization

The most commonly formulated structural topology optimization problem is to generate material layout which within a design domain leads to a minimal value of the structure compliance *c*, Equation (1). Hence, one can follow the optimization problem formulated in a widely recognized paper by Sigmund [26]. The available material volume fraction *κ* is defined and treated in the optimization process as the constraint imposed on structure volume *V*, Equation (2). The finite element approach has been applied:

(1) $$\mathrm{minimize}\;c\left(d\right)=u^{T}ku=\overset{N}{\underset{i=1}{\sum }}d_{i}^{p}u_{i}^{T}k_{i}u_{i}$$

(2) $$\mathrm{subject}\;\mathrm{to}\;V\left(d\right)=\kappa V_{0}$$

(3) k u=f

(4) $$0<d_{min}\leq d_{i}\leq 1.$$

The quantity $u_{i}$ denotes the displacement vector, $\;k_{i}$ stands for the stiffness matrix, and both are defined for *N* elements. The design variable $d_{i}$, which represents the relative material density, is assigned to each element. In Equation (3), k  represents the global stiffness matrix, u stands for the global displacement vector, and f  is the vector of forces. Due to the simple bounds imposed in Equation (4) on the design variables with $d_{min}$ (e.g., 10^−9^) as a non-zero minimum value of relative density, singularity is avoided.

The SIMP (solid isotropic material with penalization) approach (e.g., [27]) in the form of power law is adapted as the material representation, see Equation (5). The modulus of elasticity $E_{i}\;$ for each finite element is a function of the design variable $d_{i}$:

(5) $$E_{i}=d_{i}^{p}E_{0}.$$

In Equation (5), the quantity $E_{0}$ stands for modulus of elasticity, defined for a solid material, whereas *p* (typically *p* = 3) is responsible for penalization of intermediate densities. This allows controlling the design process and leads to obtaining black-and-white resulting structures. During the topology generation process, a material is redistributed within the design domain, which results in removing parts unnecessary from design criteria viewpoint.

### 2.2. Concept of the Cellular Automaton Mimicking Colliding Bodies

The selection of a proper method of topology generation determines the effectiveness of the topology optimization process. Heuristic optimization techniques become popular among researchers because they are easy to implement numerically, gradient information is not required, and one can easily combine this type of algorithm with any finite element structural analysis code.

**Figure 1:**
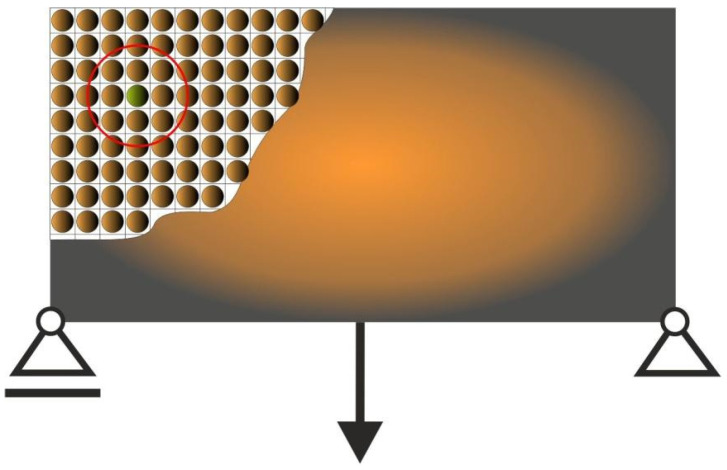
The cell lattice. A body is placed in each cell. The neighborhood, which is represented by the red circle, is identified around each body/cell.

In this paper, the original heuristic topology generator built as Cellular Automaton which mimics Colliding Bodies (CAmCB) is proposed. The idea is that the design domain of the structure is decomposed into a lattice of cells which are usually equivalent to finite elements. For each cell, the surrounding cells form a neighborhood. The bodies are distributed within this lattice (Figure 1).

**Figure 2:**
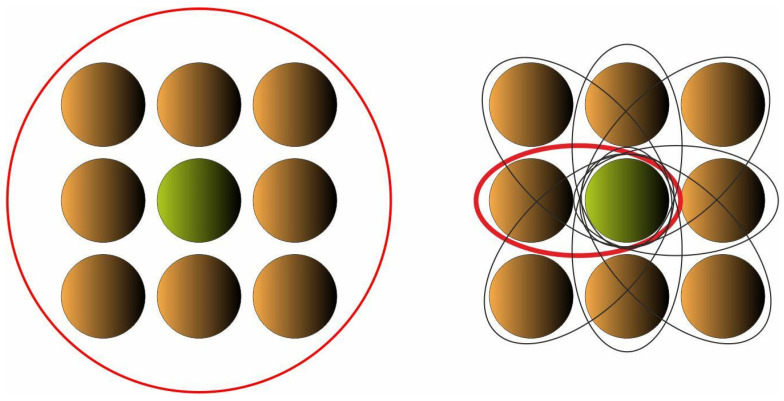
The cell neighborhood. The neighboring cells collide with the central one.

Let us assume that the bodies have masses and velocities. Masses are proportional to cell areas whereas velocities are related to cell compliances. Furthermore, let us imagine that the neighboring bodies collide with the central one, which results in changing its status. In what follows, the central body can either be forced to remain in its position, or is pushed away (Figure 2).

**Figure 3:**
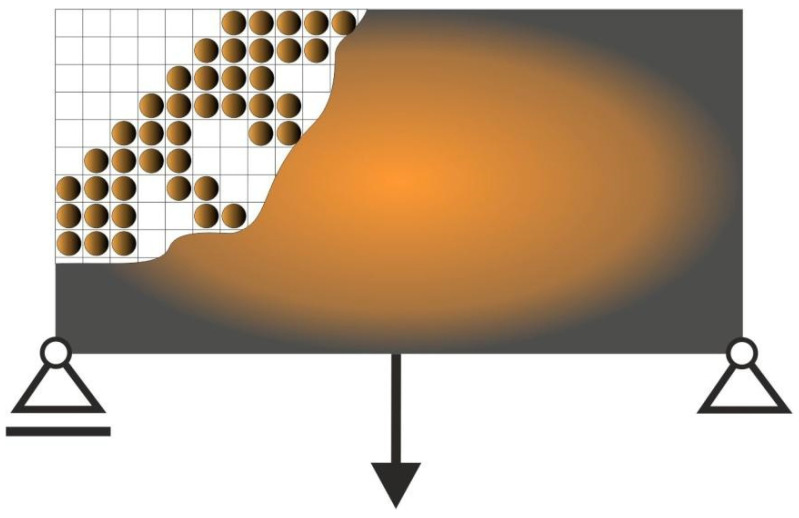
The topology generation. Some cells remain, some are eliminated.

From a topology generation point of view, the interpretation is that the central cell remains solid, or is driven to become a void one (Figure 3).

### 2.3. Local Update Rules

**Figure 4:**
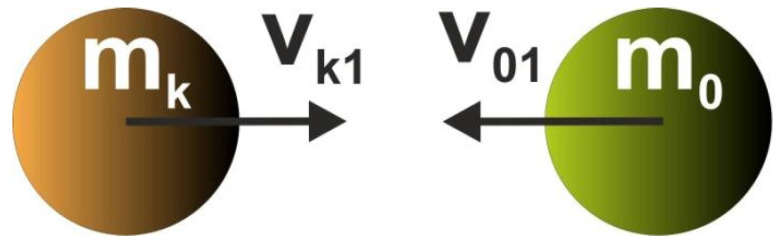
A neighboring body (k) collides with the central one (0). Before collision.

**Figure 5:**
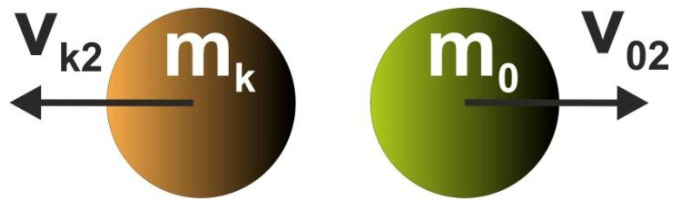
A neighboring body (k) collides with the central one (0). After collision.

While building Cellular Automaton, it is assumed that the interactions between bodies/cells take place only within the specified neighborhood, where they are governed by local rules which are identical for all cells, and are applied simultaneously to each of them. According to the concept of the paper, the local rules are derived so as to mimic collisions taking place between bodies/cells within each neighborhood. The governing equations based on the physics laws of momentum and energy are applied. Let us consider the central cell and a neighboring one colliding with it (Figure 4 and Figure 5).

The governing equations are as follows:

(6) $$m_{k}v_{k1}-m_{0}v_{01}=-m_{k}v_{k2}+m_{0}v_{02}$$

(7) $$\frac{1}{2}m_{k}v_{k1}^{2}+\frac{1}{2}m_{0}v_{01}^{2}=\frac{1}{2}m_{k}v_{k2}^{2}+\frac{1}{2}m_{0}v_{02}^{2}.$$

Based on the above, the velocity of the central cell after collision equals:

(8) $$v_{02}=\frac{\left(m_{k}-m_{0}\right)v_{01}+2m_{k}v_{k1}}{m_{0}+m_{k}}.$$

As stated earlier, velocities are related to cell compliances and masses are proportional to cell areas. Equation (8) can be rewritten in the form of Equation (9):

(9) $$F_{02}=\frac{\left(A_{k}-A_{0}\right)F_{01}+2A_{k}F_{k1}}{A_{0}+A_{k}},$$

where A represents the cell area and F is a function associated with local compliances. It is proposed to select the quantity $F_{02}$ as the basis for building the update rule. Before that, the details regarding how to calculate F values are given.

Based on the results obtained from a structural analysis, the values of local compliances are calculated for all cells/elements. The compliances are sorted then in the ascending order, and those having the lowest and the highest values are identified. In the next step, $N_{1}$, $N_{2}$ are selected and values of F  are assigned to cells $\left(i=1,2\ldots n\right)\;$ according to Equation (10):

(10) $$F_{i}=\left\{\begin{array}{c} -C\;\;\;\mathrm{if}\;\;i<N_{1}\\ \;\;\;f_{i}\;\;\;\mathrm{if}\;\;\;\;N_{1}\leq i\leq \\ \;\;C\;\;\;\;\mathrm{if}\;\;i>N_{2} \end{array}N_{2}.\right.$$

A monotonically increasing function representing cell compliances is selected for the intermediate interval $N_{1}\leq i\leq N_{2}$ and then function values are assigned to the cells, respectively. Here, the linear function is selected to fulfill: $\;f_{i}(N_{1})=-C\;$ and $f_{i}(N_{2})=C$, thus:

(11) $$f_{i}=2C\frac{i}{N_{2}-N_{1}}-C\frac{N_{2}+N_{1}}{N_{2}-N_{1}}.$$

The quantity C  in Equation (11) is a user-specified parameter, usually equal to 1. The above described compliance mapping technique, represented by Equations (10) and (11), has been discussed also in [25].

Having finished with data preparation, the update rule can be built. Hence, repeating collisions for all neighboring cells, the average quantity $F_{02}$ can be calculated based on Equation (12):

(12) $${\bar{F}}_{02}=\frac{1}{M}\overset{M}{\underset{k=1}{\sum }}F_{02\left(k\right)}.$$

Finally, the design variables can be updated according to Equation (13):

(13) $$d_{new}^{\left(i\right)}=d^{\left(i\right)}+m{\bar{F}}_{02},$$

where  m denotes the move limit (e.g., m=0.2).

### 2.4. Algorithm

In order to implement the above-proposed design rule, a numerical algorithm was built. The sequential approach was adapted for the optimization procedure, meaning that for each iteration, the structural analysis performed for the optimized element is followed by a local updating process. Simultaneously, for a specified volume fraction, a global volume constraint is applied. As a result, during the optimization process, the generated topologies preserve a specified volume fraction of a solid material.

The issue to discuss regards the form of Equation (9). In the case of a regular lattice of cells/elements, the first component of the numerator vanishes. In order to preserve the influence of the central cell compliance on the final result during the iteration process, it is proposed to modify the cells area representation:

(14) $$A_{i}=A\left[1+b\left(2r-1\right)\right],$$

where

(15) $$b=b_{0}\left(1-\frac{t-1}{t_{max}-1}\right).$$

In Equations (14) and (15), $b_{0}$ is a small value, r is a random number taken from [0, 1] interval, t stands for the current iteration number, whereas $t_{max}$ is a selected number of iterations. As a result, $A_{i}=A$ only for $t=t_{max}$.

In order to control the topology generation, the threshold values $N_{1}$ and $N_{2}$ can be modified so as to adjust the width of the interval [$N_{1}$, $N_{2}$] during the iteration process. It is proposed to start with a relatively wide interval, and then to reduce it successively. As a result, at the beginning of the topology generation process, the large design domain is searched by the Automaton, and the majority of void cells is eliminated. Then, during the iterative process while reducing the interval [$N_{1}$, $N_{2}$], the so-called gray cells of intermediate densities are eliminated, which finally results in obtaining distinct solid/void structures.

### 2.5. Introductory Example

**Figure 6:**
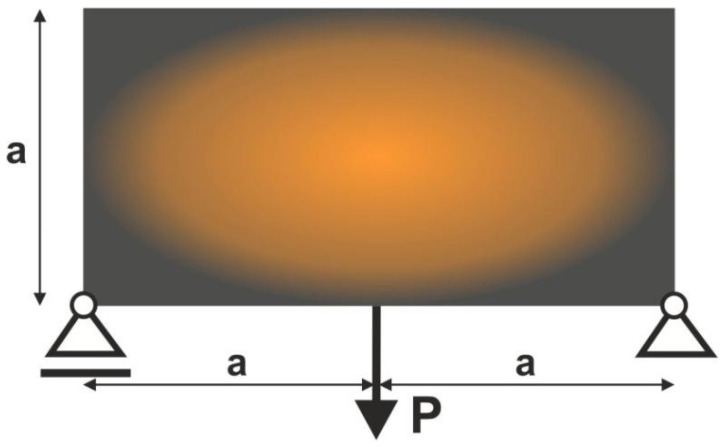
The rectangular structure with applied load and support.

The rectangular structure shown in Figure 6 has been chosen as the introductory example. The mesh of 3200 (80×40) square elements/cells has been generated to perform structural analysis and topology optimization for the data: $E_{0}=10\;\mathrm{GPa},\;\nu =0.3,\;P=100\;N,\;a=40\;\mathrm{mm},\;\kappa =0.5,\;b_{0}=0.05$. As for the topology generation, the Moore type neighborhood, i.e., cells having common vertices with the central one, has been applied.

**Figure 7:**
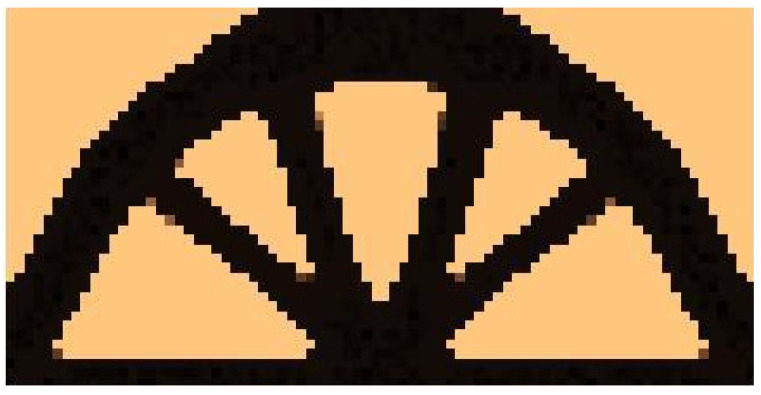
The final topology.

**Figure 8:**
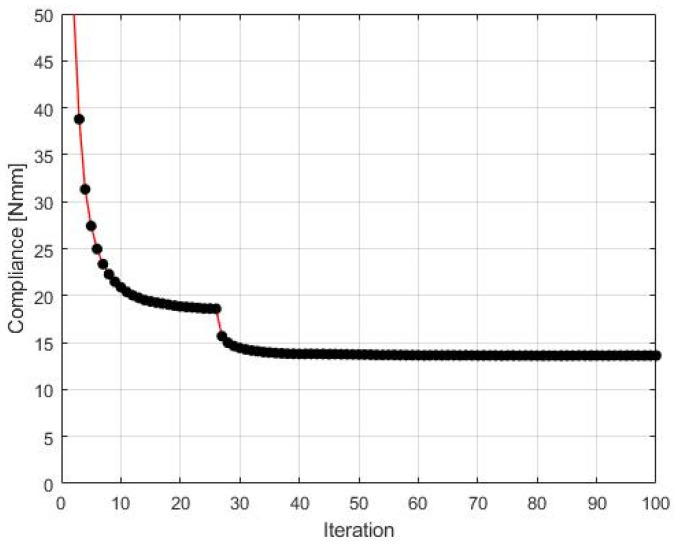
The compliance history. Minimal value: 13.62 Nmm. Black dots on the red line represent the compliance values for subsequent iterations.

The CAmCB algorithm found the final topology, which is shown in Figure 7, whereas the iteration history is given in Figure 8. The strategy of $F_{i}$, see Equation (10), implementation was as follows: one started with $N_{1}=N\cdot 0.02$, and then from iteration 25 $N_{1}=N\cdot 0.5$, where N is the number of cells. Simultaneously, $N_{2}=N\cdot 0.6\;$ has remained fixed for the entire iteration process.

The compliance value found for this structure is equal to 13.62 Nmm. This outperforms the solution reported in [25] where compliance of 14.02 Nmm has been obtained for the final topology.

## 3. Results and Discussion

The original examples of topology generation are discussed in this section, presenting the performance of the algorithm. With a view to cover a broad area of implementations, the discussed tasks regard plane and spatial structures. The case of irregular cell lattice is also considered.

In what follows, to illustrate more thoroughly how the proposed CAmCB algorithm works, some numerical examples have been selected. The algorithm performance is presented first for plane test structures, and then for plane and spatial engineering structures. For the test structures, a Matlab-based algorithm has been applied, whereas for engineering structures, the topology generator has been combined with the ANSYS system, which was responsible for performing the structural analysis.

### 3.1. Topology Generation for the Test Structures

The results of topology generation performed for four plane test structures are presented below.

#### 3.1.1. Test Structure 1

**Figure 9:**
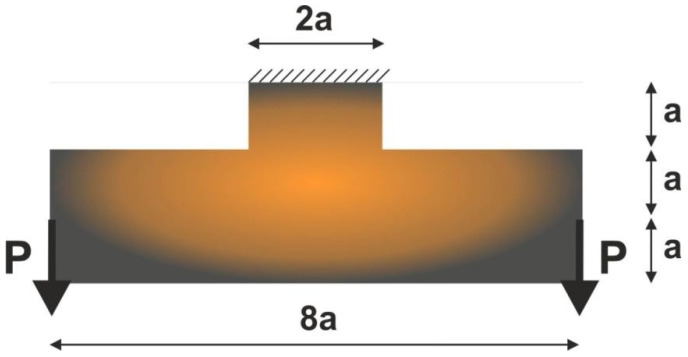
The test structure 1 with applied loads and support.

To perform the first test, the structure shown in Figure 9 has been selected. The mesh of 60,000 (400×150) square elements/cells has been implemented, and structural analysis and topology optimization have been performed for the data: $E_{0}=10\;\mathrm{GPa},\;\nu =0.3,\;P=100\;N,\;a=50\;\mathrm{mm},\;\kappa =0.25,\;b_{0}=0.05$. The Moore type neighborhood has been applied.

**Figure 10:**
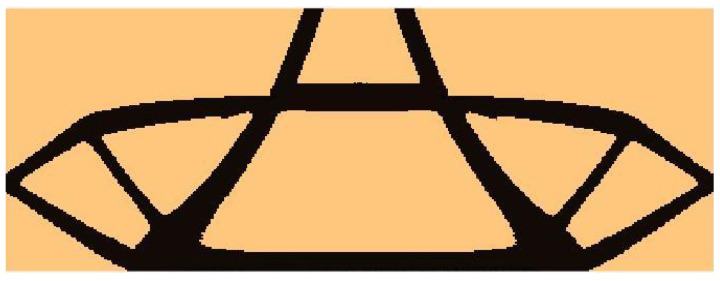
The final topology found for the test structure 1.

**Figure 11:**
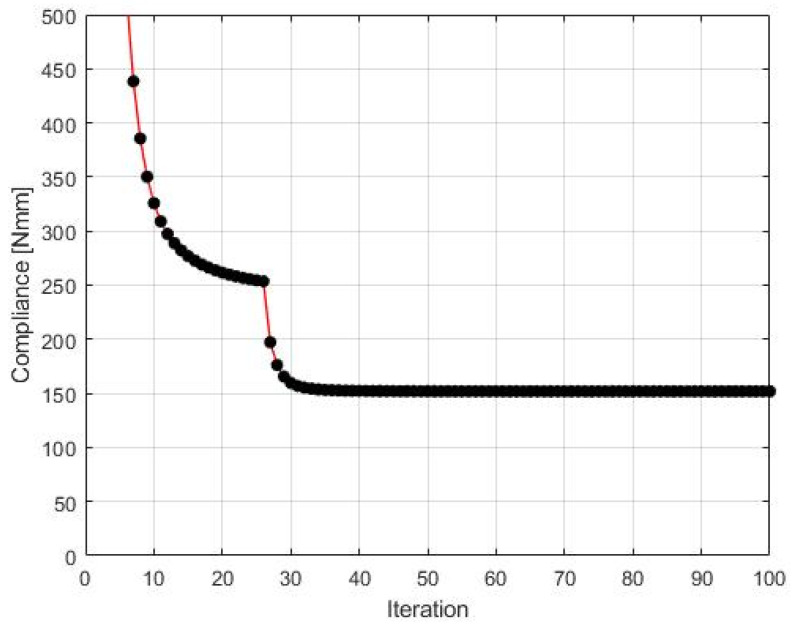
The compliance history for the test structure 1. Minimal value: 151.84 Nmm. Black dots on the red line represent the compliance values for subsequent iterations.

The algorithm found the final topology, which is shown in Figure 10, whereas the iteration history is given in Figure 11. The strategy of $F_{i}$ implementation was as follows: one started with $N_{1}=N\cdot 0.02$, and then from iteration 25 $N_{1}=N\cdot 0.75$, and from iteration 75 $N_{1}=N\cdot 0.9$. The quantity $N_{2}=N\cdot 0.98\;$ has remained fixed for the whole iteration process.

#### 3.1.2. Test Structure 2

**Figure 12:**
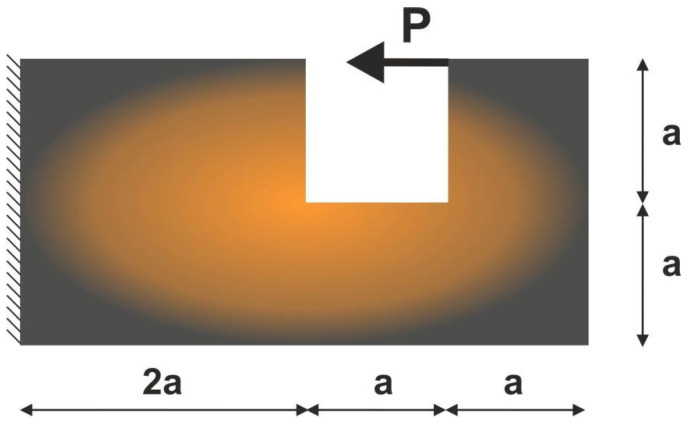
The test structure 2 with applied load and support.

For the structure shown in Figure 12, the mesh of 80,000 (400×200) square elements/cells has been generated. The structural analysis and topology optimization have been performed for the data: $E_{0}=10\;\mathrm{GPa},\;\nu =0.3,\;P=100\;N,\;a=100\;\mathrm{mm},\;\kappa =0.3,\;b_{0}=0.05$. The Moore type neighborhood has been applied.

**Figure 13:**
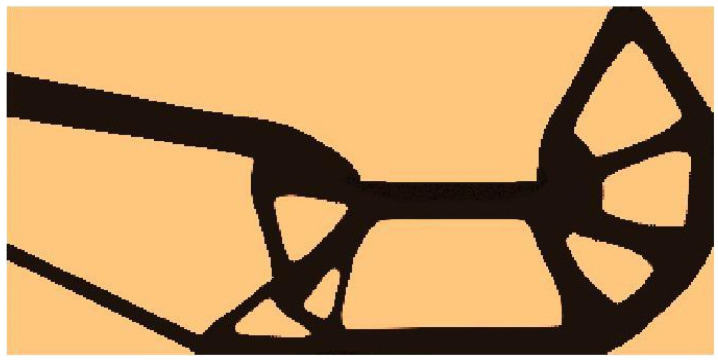
The final topology found for the test structure 2.

**Figure 14:**
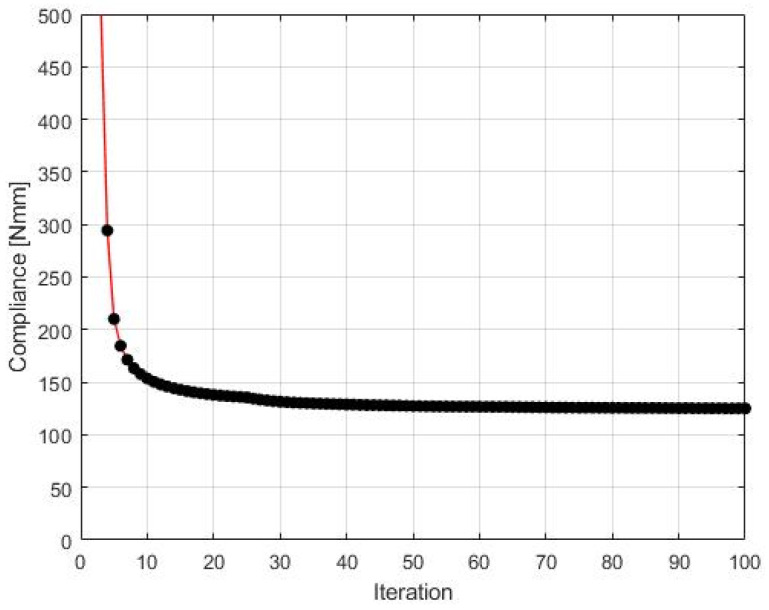
The compliance history for the test structure 2. Minimal value: 125.07 Nmm. Black dots on the red line represent the compliance values for subsequent iterations.

The final structure topology found by the algorithm and the illustration of the compliance history are given in Figure 13 and Figure 14, respectively. As for the strategy of implementation of $\;F_{i}$, one started with $N_{1}=N\cdot 0.35,$ and then from iteration 25 $N_{1}=N\cdot 0.5$, from iteration 50$\;N_{1}=N\cdot 0.75$, and finally from iteration 75 $N_{1}=N\cdot 0.9.\;N_{2}=N\cdot 0.98\;$ has remained fixed for all iterations.

#### 3.1.3. Test Structure 3

**Figure 15:**
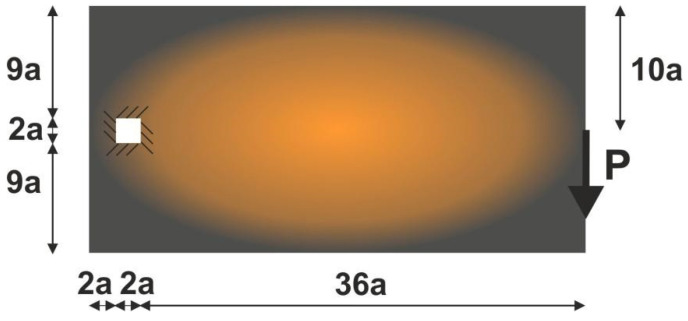
The test structure 3 with applied load and support.

To perform the third test, the structure shown in Figure 15 has been proposed. The mesh of 80,000 (400×200) square elements/cells has been implemented and structural analysis and topology optimization have been performed for the data: $E_{0}=10\;\mathrm{GPa},\;\nu =0.3,\;P=100\;N,\;a=10\;\mathrm{mm},\;\kappa =0.3,\;b_{0}=0.05$. The Moore type neighborhood has been applied.

**Figure 16:**
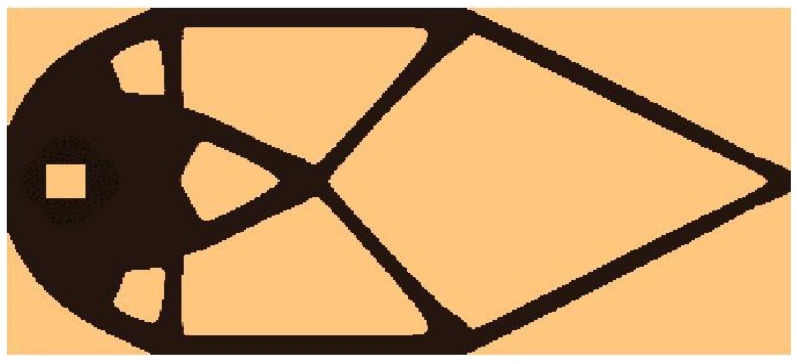
The final topology found for the test structure 3.

**Figure 17:**
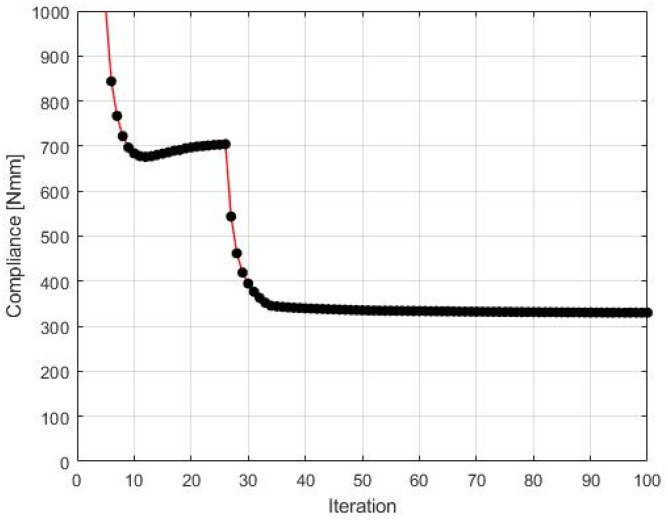
The compliance history for the test structure 3. Minimal value: 342.96 Nmm. Black dots on the red line represent the compliance values for subsequent iterations.

The algorithm found the final topology, which is shown in Figure 16, whereas the iteration history is given in Figure 17. The strategy of $F_{i}$ implementation was as follows: one started with $N_{1}=N\cdot 0.02,$ and then from iteration 25 $N_{1}=N\cdot 0.75$, from iteration 50 $N_{1}=N\cdot 0.75$, and finally from iteration 75 $N_{1}=N\cdot 0.9$. The quantity $N_{2}=N\cdot 0.98$ remained fixed for the whole iteration process.

#### 3.1.4. Test Structure 4

**Figure 18:**
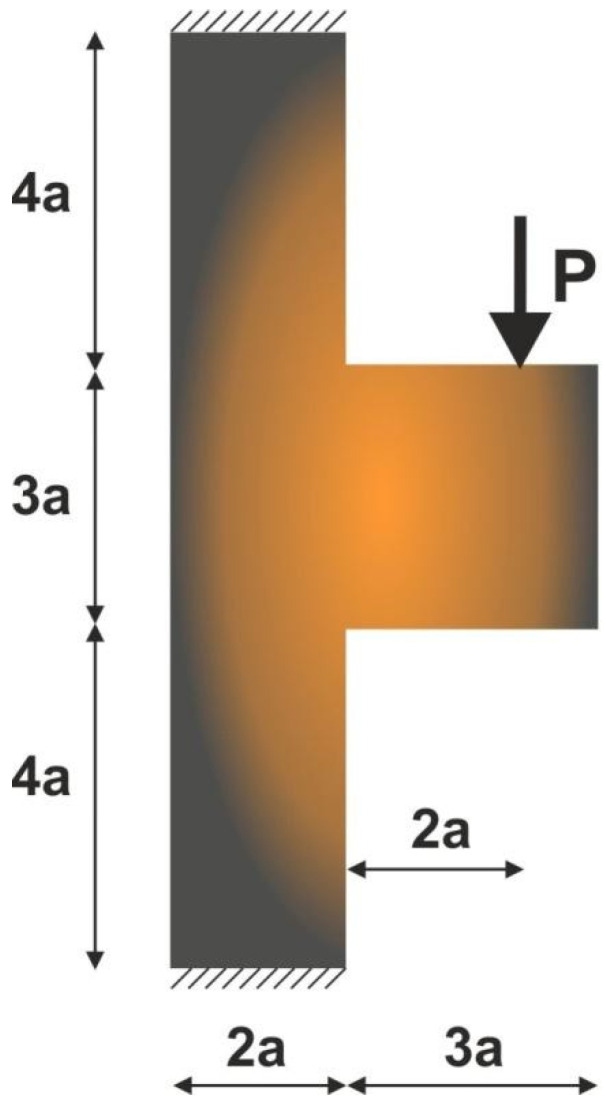
The test structure 4 with applied load and support.

For the structure shown in Figure 18, the mesh of 137,500 (250 × 550) square elements/cells has been applied. The structural analysis and topology optimization have been performed for the data: $E_{0}=10\;\mathrm{GPa},\;\nu =0.3,\;P=100\;N,\;a=50\;\mathrm{mm},\;\kappa =0.25,\;b_{0}=0.05$. The Moore type neighborhood has been applied.

**Figure 19:**
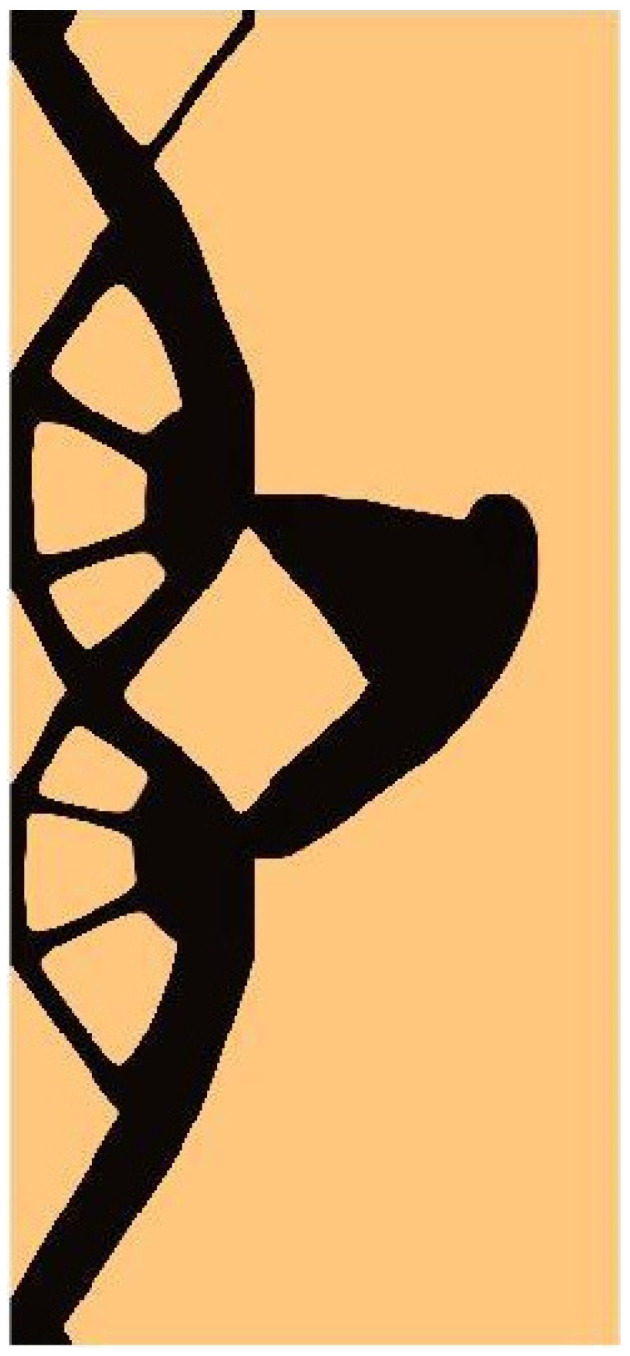
The final topology found for the test structure 4.

**Figure 20:**
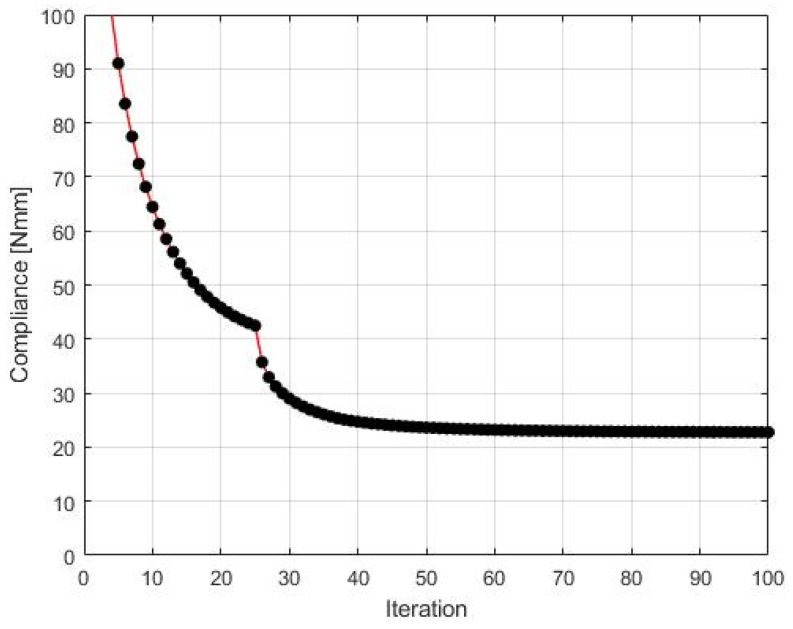
The compliance history for the test structure 4. Minimal value: 22.71 Nmm. Black dots on the red line represent the compliance values for subsequent iterations.

The final structure topology found by the algorithm and the illustration of the compliance history are given in Figure 19 and Figure 20, respectively. As for the strategy of $F_{i}$ implementation: one started for $N_{1}=N\cdot 0.02$, and then from iteration 25 $N_{1}=N\cdot 0.5$, from iteration 50 $\;N_{1}=N\cdot 0.75$, and finally from iteration 75 $N_{1}=N\cdot 0.9.\;N_{2}=N\cdot 0.98$ has remained fixed for all iterations.

As can be seen from the above, the original CAmCB algorithm can effectively generate minimal compliance topologies. It is also worth comparing the obtained results with the ones which can be found for the considered structures when using other existing and popular approaches. The top88 algorithm [28] based on the optimality criterion and the PTOc one [29], utilizing the concept of proportional topology optimization have been selected for this purpose. The above papers provide Matlab codes of topology generators and these have been used to perform computations for the test structures defined earlier in this section. Table 1 gathers the results of these computations.

One can observe that the CAmCB algorithm proposed in this paper allows us to find results which can be better in terms of objective function values than the ones obtained with the use of other approaches selected for this comparison.

### 3.2. Engineering Applications

A series of illustrative engineering examples has been selected to examine the effectiveness of the introduced concept of the CAmCB topology generator. Both regular and irregular cell lattices are considered to show the algorithm performance and the versatility of the approach. As mentioned earlier, the proposed topology generator can be easily combined with any solver built on finite element methods. Hence, the optimization module has been linked to the professional system ANSYS to perform structural analyses. It is worth noting that the proposed algorithm does not require additional density filtering, the so-called gray elements are eliminated, and the checkerboard effect has not been observed for generated topologies.

#### 3.2.1. Mechanical Part

**Figure 21:**
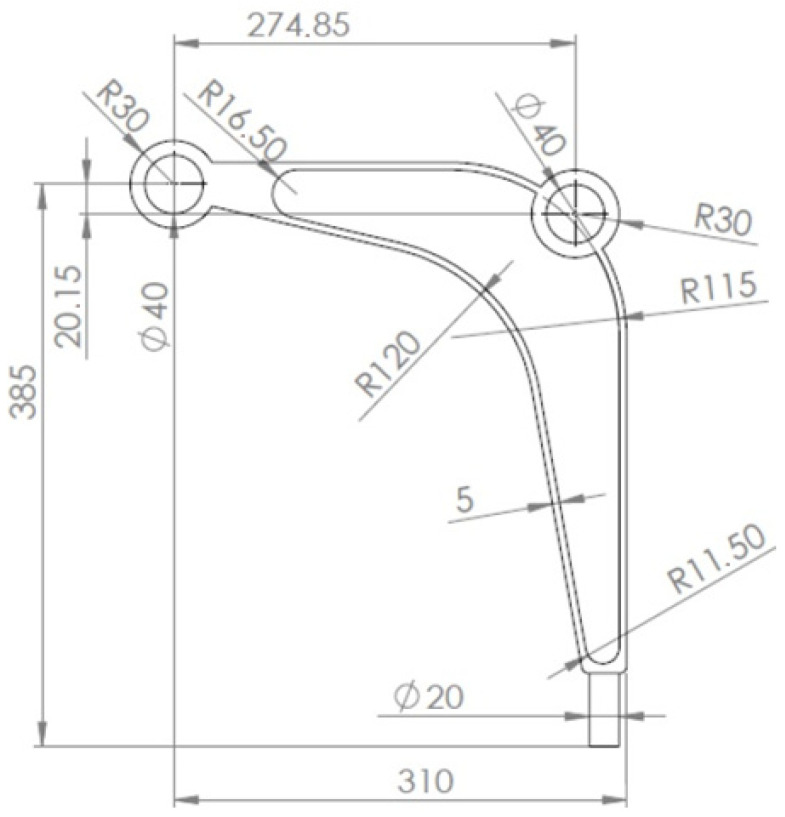
The control arm: dimensions in mm.

**Figure 22:**
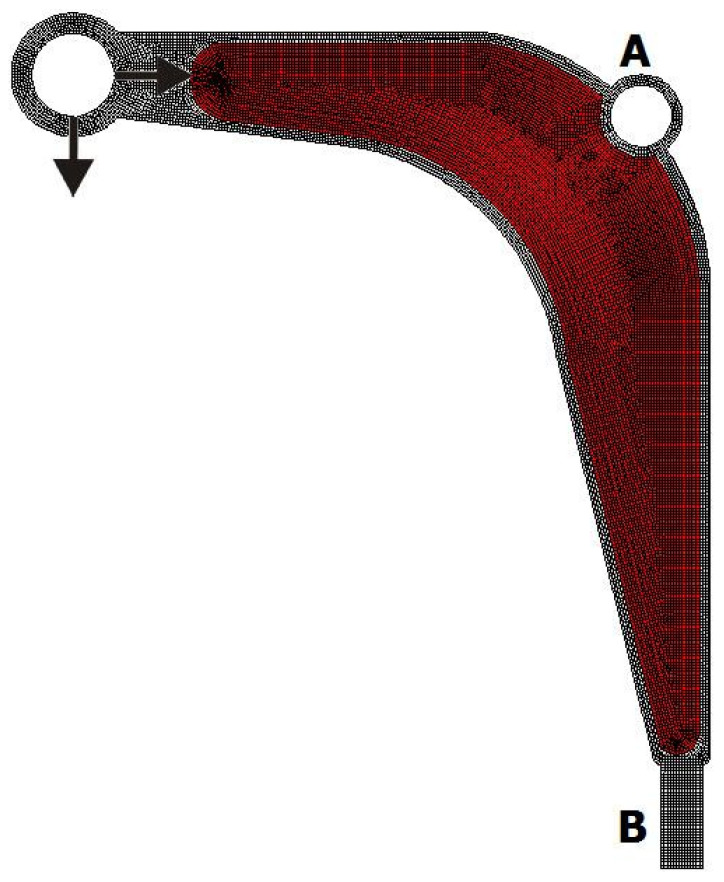
The design domain, loads, and supports of the control arm.

The model of a control arm structure presented in Figure 21 has been chosen for this purpose. The mesh of 16,304 elements/cells has been generated to perform structural analysis and topology optimization for the data: $E_{0}=210\;\mathrm{GPa},\;\nu =0.28,\kappa =0.4,\;b_{0}=0.01$. The structure consists of a non-optimized region presented in Figure 22 as a gray area whereas the design domain is presented as a red area. The structure is loaded by two concentrated forces: a horizontal force equal to 7000 N and a vertical one equal to 2700 N. The horizontal displacement of nodes in the inner bound of the round hole A are equal to zero, while all nodes in area B are fixed.

As for the strategy of $F_{i}$ implementation: one starts with $N_{1}=N\cdot 0.02$, and then from iteration 25 $N_{1}=N\cdot 0.5$, and from iteration 50 $N_{1}=N\cdot 0.75,\;\;\mathrm{whereas}\;N_{2}=N\cdot 0.98$ remains fixed for all iterations. This strategy has been applied for all presented engineering examples. It is worth pointing out that in order to complete the optimization process about 50 iterations are needed.

**Figure 23:**
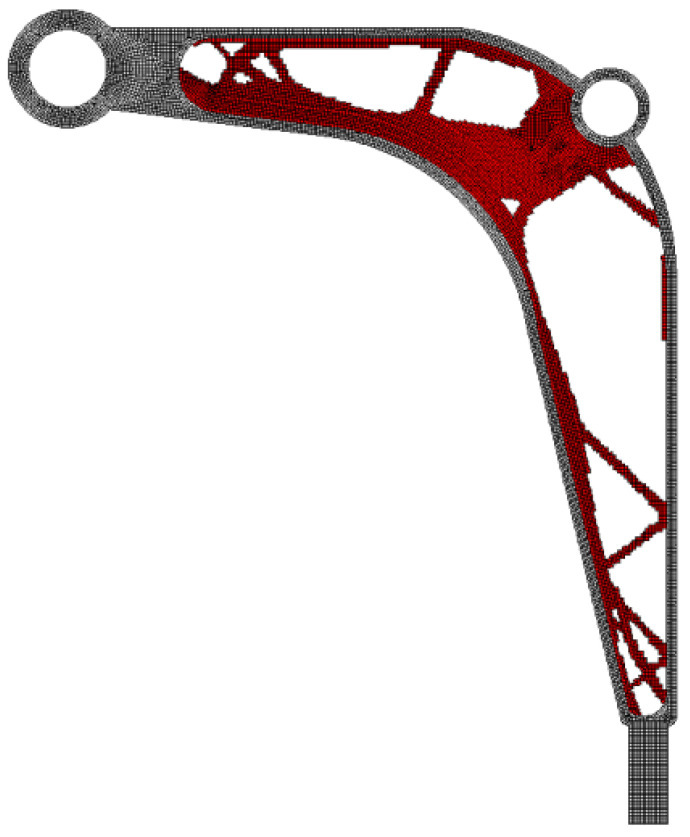
The final topology found for the control arm.

The algorithm found the final topology, which is shown in Figure 23. The resulting compliance equals 11,949 Nmm. Referring to the prior comparison of the results, the value of 12,372 Nmm was obtained when the algorithm [28] was utilized.

The CAmCB algorithm codes for the example considered in this section are provided in the Supplementary Files.

#### 3.2.2. The Frame Structure-Generation of Topology for Irregular Cell Lattices

The aim of this example is to extend the presentation of the proposed algorithm toward an irregular grid of cells related to a non-regular mesh of finite elements. Resizing a traditional uniform grid of cells allows us to obtain flexible solutions, for e.g., extremely irregular design domains where it is difficult or impossible to cover them with uniform cells. Additionally, regions with stress concentrations, such as around holes or sharp edges, should be covered with a fine mesh, which is not necessary for the structure as a whole. The procedure of refining a mesh in selected regions can be used in order to achieve an accurate solution without an excessive increase of the number of elements caused by using a fine mesh implemented for the whole structure.

**Figure 24:**
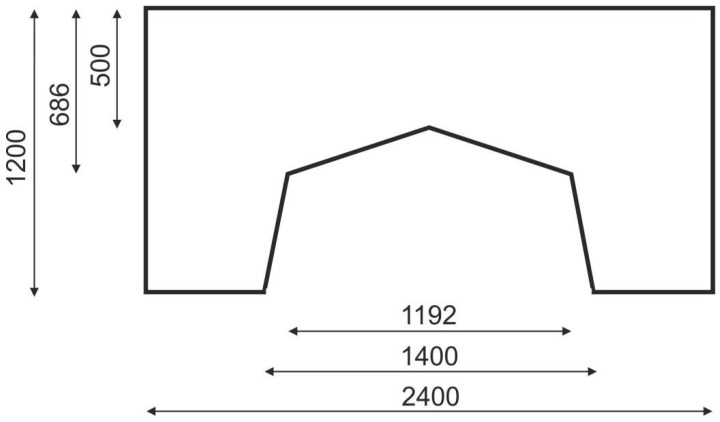
The portal frame: dimensions in mm.

**Figure 25:**
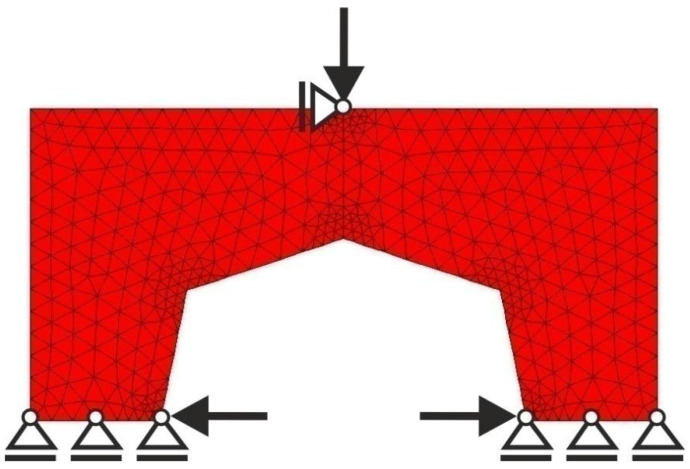
The design domain, loads, and supports of the portal frame.

**Figure 26:**
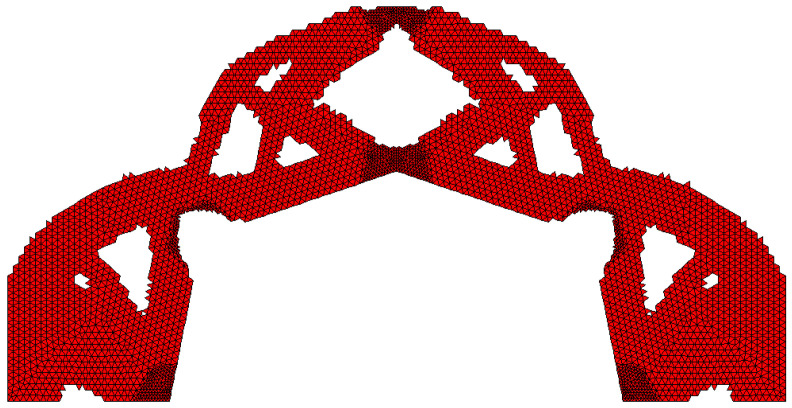
The final topology found for the portal frame.

The example illustrating this case is the portal frame presented in Figure 24. The data is as follows: $E_{0}=200\;\mathrm{GPa},\;\nu =0.25,\;\kappa =0.5,\;b_{0}=0.01$. The irregular lattice of cells is distributed according to Figure 25. For the irregular lattice of 14,024 cells (two-dimensional 6-node triangular elements—Plane82) ANSYS software was utilized for static analysis in the optimization process. The optimization has been performed and the obtained final topology is presented in Figure 26. Loads of 100 N each have been applied. The resulting compliance is equal to 5.03 × 10^−3^ Nmm.

The algorithm found the final topology, which is shown in Figure 26.

#### 3.2.3. The Box Tube-Generation of Topology for Spatial Structure

**Figure 27:**
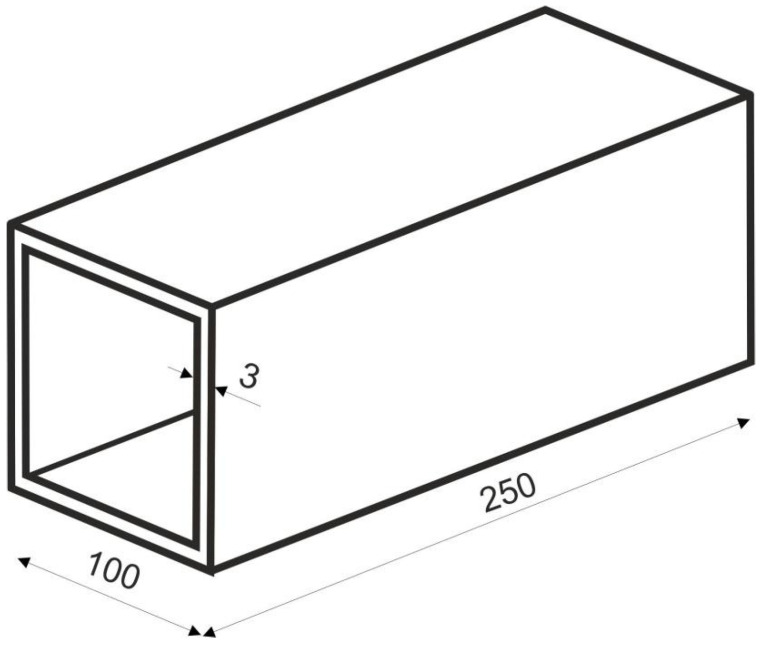
The box tube: dimensions in mm.

**Figure 28:**
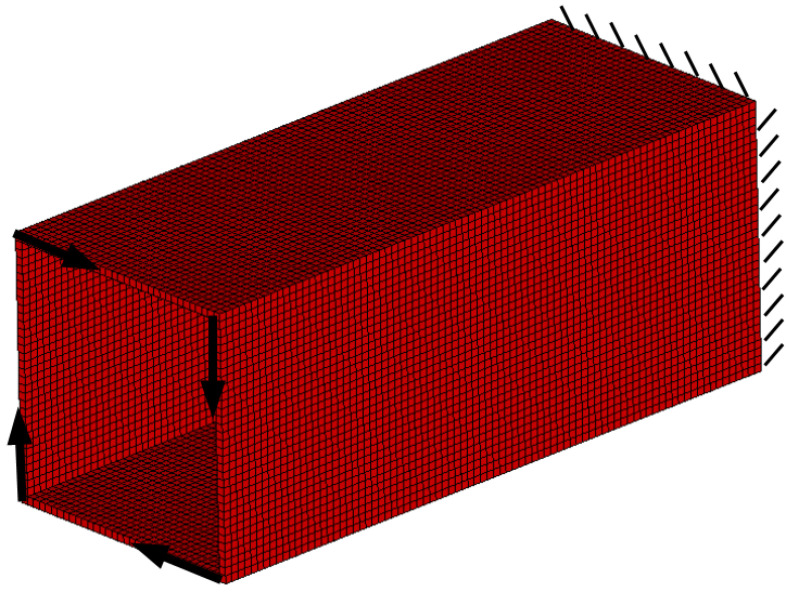
The design domain, loads, and supports of the box tube.

**Figure 29:**
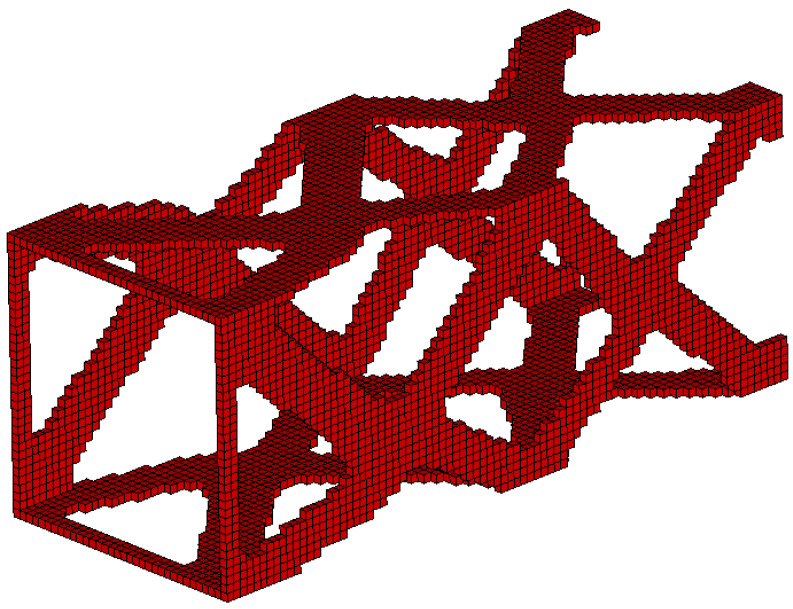
The final topology for the box tube for volume fraction κ=0.4.

The box tube shown in Figure 27 has been selected as the final example. The box tube cross section with 3 mm wall thickness is a square (100 mm×100 mm), the tube is 250 mm long. Loads of 1000 N each have been applied as shown in Figure 28. The data is as follows: $E_{0}=200\;\mathrm{GPa},\;\nu =0.3,\;\kappa =0.4,\;b_{0}=0.01$. A regular mesh of 11,088 three-dimensional 8-node elements (Solid45) has been applied for a static analysis made by ANSYS software (the length of the element edge is 3 mm). For the example of this section, the algorithm utilizes the von Neumann type of neighborhood. The resulting topology is presented in Figure 29, for which the final compliance equals 278.8 Nmm.

The algorithm found the final topology which is shown in Figure 29.

**Figure 30:**
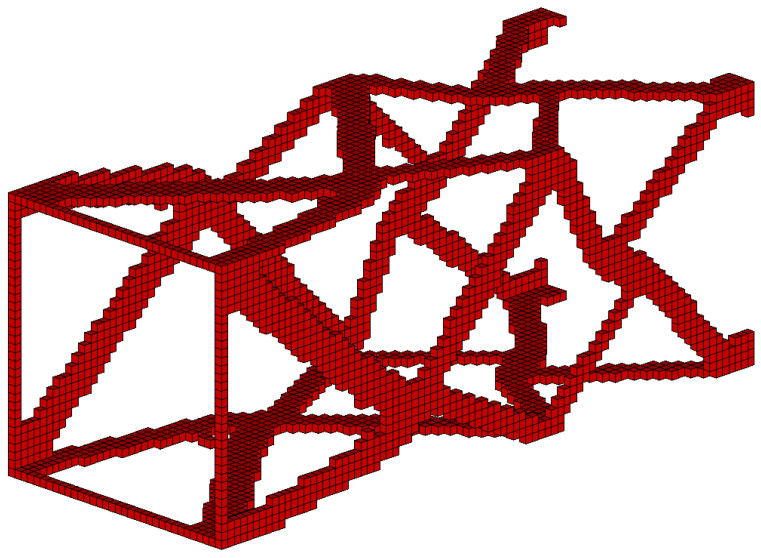
The final topology for the box tube for volume fraction κ=0.25.

The algorithm performance was additionally tested based on the same example, repeating computations for low volume fraction κ=0.25. The resulting topology for which the final compliance reaches the value equal to 586.3 Nmm is presented in Figure 30.

## 4. Concluding Remarks

The discussion regarding the proposed algorithm and its performance is summed up in this section. In the presented study, the original concept of Cellular Automaton mimicking Colliding Bodies (CAmCB) has been applied for topology optimization using the minimum compliance as the objective function. The CAmCB algorithm combines Cellular Automata heuristic with Colliding Bodies phenomenon to create a fast convergent technique which provides black-and-white topologies, without gray regions and the checkerboard effect. Moreover, additional density filtering is not necessary and there is no need to calculate gradients. In order to illustrate the effectiveness of the proposed CAmCB algorithm, selected numerical examples have been investigated. The algorithm performance is presented for plane test structures and for plane and spatial engineering structures. In the latter case, the proposed optimizer was combined with professional FEM analysis codes. The advantage of the developed algorithm is that it is a versatile technique which allows implementation of rectangular or triangular lattices, adaptation to highly non-uniform finite element lattices, as well as consideration of the total volume constraint with large and small volume fraction which is important especially for lightweight topology optimization. Preliminary studies reveal the possibility of applying CAmCB algorithm into uncommon but interesting issues such as the consideration of design-dependent loading (self-weight) or topology optimization of multi-material structures. The results of the tests performed so far are encouraging, which allows us to consider the proposed concept of the automaton mimicking colliding bodies phenomenon as an alternative algorithm to other existing topology generators suited for engineering applications.

## Figures and Tables

**Table 1:** Comparison of minimum compliance values [Nmm] found for the three algorithms.

Algorithm	Test Structure 1	Test Structure 2	Test Structure 3	Test Structure 4
CAmCB	151.84	125.07	342.96	22.71
top88 [28]	164.26	139.91	360.90	23.84
PTOc [29]	164.90	127.49	347.93	23.93

## Data Availability

The data presented in this study are available on request from the corresponding author.

## Supplementary Materials

The following supporting information can be downloaded at: https://www.mdpi.com/article/10.3390/ma15228057/s1.
